# COVID-19 – Inequities, the third wave and vaccination

**DOI:** 10.4102/safp.v63i1.5375

**Published:** 2021-08-24

**Authors:** Indiran Govender

**Affiliations:** 1 Department of Family Medicine, Faculty of Health Sciences, University of Pretoria, Pretoria, South Africa

Severe acute respiratory syndrome coronavirus 2 (SARS-CoV-2) will continue to replicate in humans, mutations will continue to occur, and variants of concern will continue to emerge. The emergence of variant strains is arguably the greatest threat to the control of the coronavirus disease 2019 (COVID-19) pandemic. A well-coordinated global strategy in prevention and control is needed. Global investments in vaccine science and technology must be accompanied by investments in public health, genomic and disease surveillance, and programmatic immunisation infrastructure to mitigate the devastating effects of COVID-19 and future pandemics.

From a public health perspective, limiting viral transmission through masking, social distancing, and other control measures will reduce new viral mutations. With continued uncontrolled transmission and viral replication, mutations that give the virus a fitness advantage are emerging. From early in the pandemic, South Africa has been a global leader in the use of intensive genomic sequencing to identify and track emerging SARS-CoV-2 mutations. The B.1.351 variant, first identified in South Africa, has shown evidence of increased transmissibility, a considerable reduction in neutralisation by convalescent and post vaccination serum, and significantly decreased neutralisation by monoclonal antibodies.

Vaccine developments against new variants will be more challenging going forward as data from randomised, placebo-controlled clinical trials become less common owing to enhanced availability of vaccines. A global scientific agenda that encompasses extensive genomic surveillance, detailed ‘correlate of protection’ evaluations, and robust post introduction surveillance and sequencing is necessary to measure the effect of new and current vaccines against SARS-CoV-2 variants. Countries will need to weigh the extent of circulating virus variants against rare adverse events, when considering vaccine choice. While the development and testing of second generation vaccines remain focussed on faster production timelines, the operational challenges of replacing current vaccines, manufacturing regional specific vaccines and administering booster doses must be prioritised. This highlights the need to improve local vaccine manufacturing capacity and investment in public health. There is no doubt that mass vaccination, with over 1 billion vaccine doses now administered worldwide, has tremendous benefit in reducing transmission as seen in Israel, the United Kingdom and the United States. Yet in Brazil and India, the same trend is not seen. The pandemic is gaining momentum in Africa as well despite aid from high-income countries with respect to personal protective equipment and oxygen. Vaccines are effective only when shots get into arms, and COVID-19 vaccine hesitancy in India, South Africa and elsewhere will pose a problem even if supplies are eventually increased. It is essential to utilise trusted, local frontline healthcare and social workers for vaccination information.

Leaders matter for public health. This has been clear in Brazil, India and the United States. At the time of this writing, only approximately 17% of people in Brazil and 10% of people in India have received at least one vaccine dose. The case rates and poor vaccination coverage in these countries pose not only a regional problem but also a global roadblock for curtailing the pandemic, with the emergence of new variants. The challenge facing low and middle-income countries (LMICs) is the apparent ‘capitalisation’ of vaccine supply where wealthier nations have greater purchasing powers resulting in vaccine surpluses capable of immunising their populations several times. The reluctance of patent protection waivers is testimony that vaccine production and supply is driven by profit, severely compromising vaccine supply to LMICs. International leaders must play their role in breaking down the financial barriers to equitable distribution of vaccine supply while supporting and strengthening safe vaccine manufacturing in LMICs to ensure quality and efficacy. The apparent sophisticated manufacturing platforms of both the Pfizer–BioNTech vaccine and the Moderna vaccine need to be made available to LMICs. In the interim, vaccine surpluses in high income countries need to be out sourced to pandemic ravaged poor countries as a matter of urgency.

The inequitable distribution of vaccines has allowed the virus to continue spreading. Unvaccinated populations are already at risk, especially from new variants, such as Delta (also known as B.1.617.2). Pledges are unlikely to get more vaccines to the world’s poorest people more quickly. In March, researchers projected that the world would be vaccinated in 2023. The extra pledges will be offset by restrictions on exports. The European Union (EU) and the United States both prohibit exports of some vaccines and vaccine ingredients. In February, India, where around six in 10 of the world’s vaccine doses are made, ordered the country’s manufacturers to stop exporting COVID-19 vaccines. This was a major setback. Coronavirus disease 2019 Vaccines Global Access (COVAX) has pledged to vaccinate one-fifth of the population of each LMIC by delivering 2 billion doses by the end of this year. But as of 02 July, 2021 COVAX had shipped only 95 million doses.

The global interest in heterologous prime-boost COVID-19 vaccination may be the answer to the battle against emerging mutant variants. Together with the shifting recommendations on the use of vaccines, several EU countries are advising those previously primed with AstraZeneca vaccine should now receive an alternative vaccine, for example, Pfizer vaccine as their second dose, administered according to a heterologous prime-boost schedule. Recently, two trials have helped to understand the reactogenicity and safety when different combinations of approved COVID-19 vaccines are administered as consecutive doses. However, the reactogenicity following the booster dose is greater with heterologous vaccinations as compared to their homologous counterparts but they are only short-term. To win the battle against the virus, perhaps this mix and match strategy of COVID-19 vaccination could lead us to victory.

